# BubbleDrive, a low-volume incubation chamber for acute brain slices

**DOI:** 10.1038/s41598-023-45949-9

**Published:** 2023-11-16

**Authors:** Aditi Naik, Vidar Jensen, Cecilie Bugge Bakketun, Rune Enger, Sabina Hrabetova, Jan Hrabe

**Affiliations:** 1 Department of Cell Biology, State University of New York Downstate Health Sciences University, Brooklyn, NY USA; 2 Neural and Behavioral Science Graduate Program, State University of New York Downstate Health Sciences University, Brooklyn, NY USA; 3 Letten Centre, Division of Anatomy, Department of Molecular Medicine, Institute of Basic Medical Sciences, University of Oslo, Oslo, Norway; 4 Department of Neurology, Oslo University Hospital, Oslo, Norway; 5 The Robert F. Furchgott Center for Neural and Behavioral Science, State University of New York Downstate Health Sciences University, Brooklyn, NY USA; 6 Translational Neuroscience Laboratories, Center for Biomedical Imaging and Neuromodulation, Nathan S. Kline Institute for Psychiatric Research, Orangeburg, NY USA

**Keywords:** Neuroscience, Biomedical engineering

## Abstract

Acute brain slices are a common and useful preparation in experimental neuroscience. A wide range of incubation chambers for brain slices exists but only a few are designed with very low volumes of the bath solution in mind. Such chambers are necessary when high-cost chemicals are to be added to the solution or when small amounts of substances released by the slice are to be collected for analysis. The principal challenge in designing a very low-volume incubation chamber is maintaining good oxygenation and flow without mechanically disturbing or damaging the slices. We designed and validated BubbleDrive, a 3D-printed incubation chamber with a minimum volume of 1.5 mL which can hold up to three coronal mouse slices from one hemisphere. It employs the carbogen gas bubbles to drive the flow circulation in a consistent and reproducible manner, and without disturbing the brain slices. The BubbleDrive design and construction were successfully validated by comparison to a conventional large-volume incubation chamber in several experimental designs involving measurements of extracellular diffusion parameters, the electrophysiology of neuronal and astrocytic networks, and the effectiveness of slice incubation with hyaluronidase enzyme.

## Introduction

Using brain slices as an experimental preparation necessitated the development of incubation chambers. Over the past 30 years, more than 35,000 studies utilized acute brain slices to investigate the cytoarchitecture and function of the brain (PubMed search for “brain slice”, 1993–2023; https://pubmed.ncbi.nlm.nih.gov). In some studies, acute brain slices are incubated with expensive or scarce pharmacological agents dissolved in artificial cerebrospinal fluid (ACSF) in order to manipulate cellular or extracellular components of the tissue. Examples of such agents include agonists or antagonists for various receptors, growth factors, enzymes, fluorescent dyes and caged compounds. In other studies, substances released from the acute brain slices into ACSF are collected for a later analysis. For both types of studies, low-volume incubation chambers are desirable in order to minimize the cost of applied agents, maximize the yield of released substances, or sharpen the timing of an ACSF concentration change. Here we describe the design, validation and application of a 3D-printed low-volume incubation chamber, BubbleDrive, that offers considerable advantages over the incubation chambers available commercially (https://www.autom8.com/brain-slice-incubators-overview/brain-slice-keeper-2-low-volume) or developed in-house^1^.

Although a wide range of incubation chambers has been designed, all of them need to fulfill the same basic requirements: (1) establish a homogeneous external environment for the slice, (2) deliver sufficient oxygen and nutrients to the slice, (3) provide mechanical support for the slice while preventing mechanical disturbance or injury, (4) allow for convenient access to place or retrieve the slices and to observe them during incubation, and (5) facilitate easy and efficient manipulation of the slice external environment (i.e. manipulation of the ACSF composition or addition of pharmacological and chemical agents). Such incubation chambers are excellent at maintaining the integrity and viability of the slices for many hours, typically 6–8 hours following their preparation.

Low-volume incubation chambers are essential for two types of experiments. First, if the experiment requires incubation of a slice with an expensive or rare chemical dissolved in the ACSF, they bring down the cost of the experiment. Incubating slices in 2 mL rather than 200 mL of ACSF will lower the cost of an experiment $100\times$. Second, if the experiment requires a post-incubation collection of the substances released by the slice, these are more likely to be found at a detectable concentration when released into a smaller volume. In addition, the properties of a small volume of ACSF can be altered more rapidly, sharpening the time response when required.

Despite their advantages, low-volume incubation chambers are not prevalent because of the technical difficulties brought about by miniaturizing the components. One of the most significant challenges is to maintain vigorous oxygenation of the ACSF without allowing the O_2_/CO_2_ bubbles to disturb the slices. Such bubbles cause mechanical waves in a tiny chamber and lead to undesirable movement of the slice, potentially resulting in slice injury. Another challenge is to maintain a consistent and steady flow of ACSF around the slices to sustain the oxygen and glucose supply.

In this study, we designed, validated and utilized a low-volume incubation chamber that we call BubbleDrive. Its minimum volume is approximately 1.5 mL and it can maintain up to three coronal slices of a mouse hemisphere. In this design, the O_2_/CO_2_ gas mixture has two functions: the gas not only dissolves in ACSF to provide oxygen for brain slices but its bubbles are also driving the circulation of oxygenated ACSF in the chamber. The ACSF oxygenation is spatially separated from the oxygen delivery to the slices, ensuring delivery of oxygen while preventing mechanical injury of the tissue due to movement caused by the gas bubbles.

BubbleDrive was validated by testing its ability to maintain the slices in healthy conditions. To this end, we studied the structural parameters of brain extracellular space (ECS), examined the function of neuronal and astrocytic networks, and tested the effectiveness of slice incubation with hyaluronidase enzyme.

## Results

### Design, fabrication and practical use of BubbleDrive

**Figure 1:**
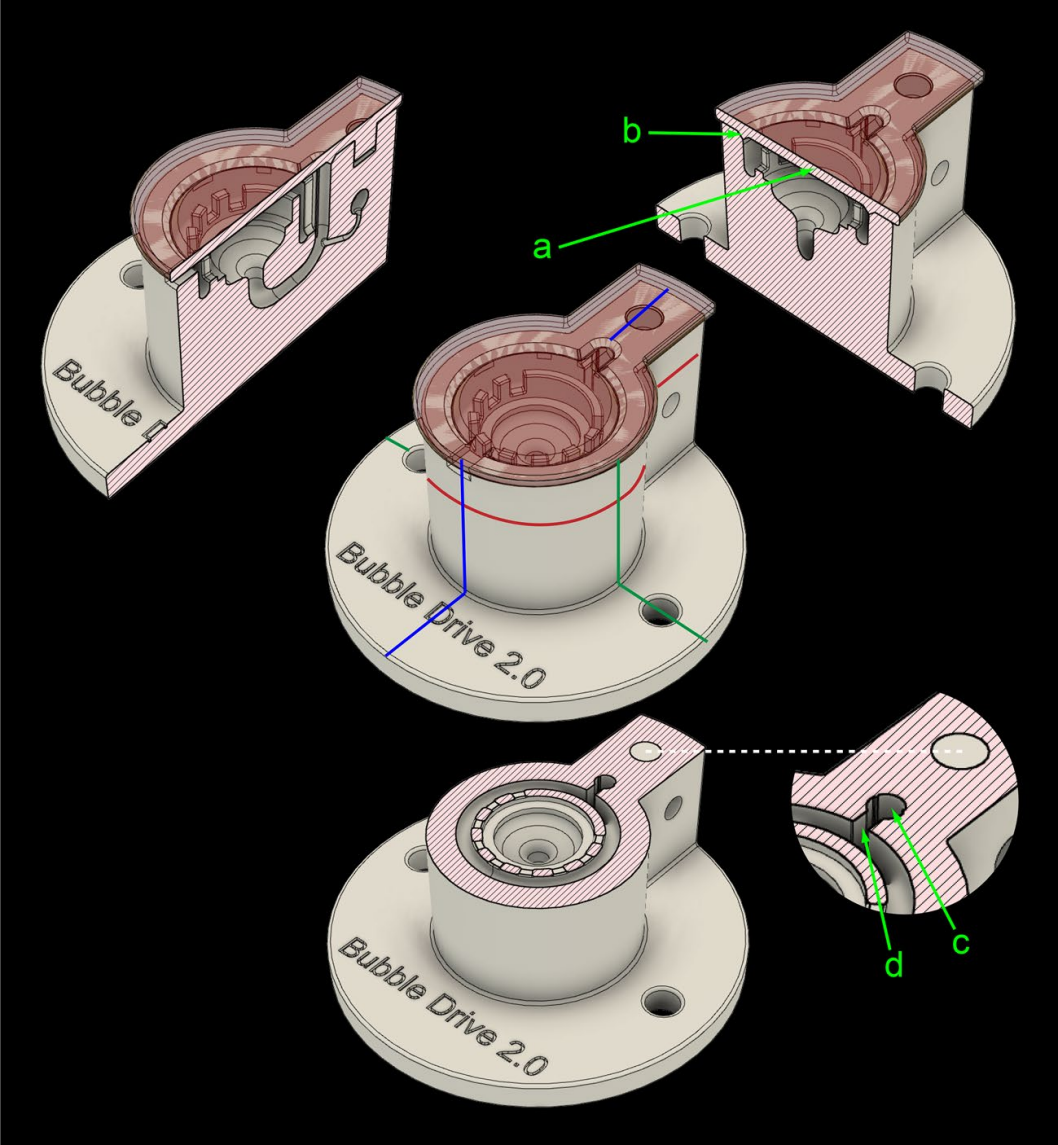
BubbleDrive model shows three cross sections along the planes marked with colored lines on the centrally positioned perspective view. (**a**) lid, (**b**) triangular lip, (**c**) chimney, (**d**) slot.

**Figure 2:**
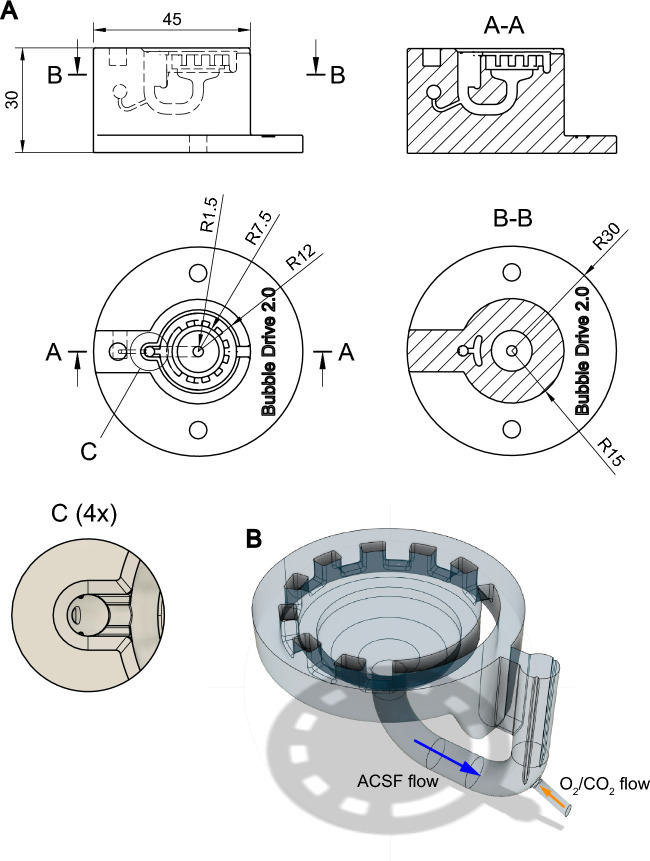
(**A**) Technical drawing of the BubbleDrive. The top and side orthographic views (middle left and upper left placements in the figure, respectively) are complemented with two cross-sectional orthographic views marked A-A and B-B, and one detailed view (4x magnification) marked (**C**). All dimensions (linear and radii) are in mm. (**B**) A 3D model of the ACSF volume inside the BubbleDrive. The direction of gas supply is marked with an orange arrow while the direction of ACSF circulation is marked with a blue arrow. The gas bubbles rise to break the surface at the top of the chimney while the heavier ACSF liquid is forced sideways through the adjacent slot and into the circular chanel. This channel is quite deep near the slot but gradually shallows further away from it to keep the overall liquid volume as small as possible.

**Figure 3:**
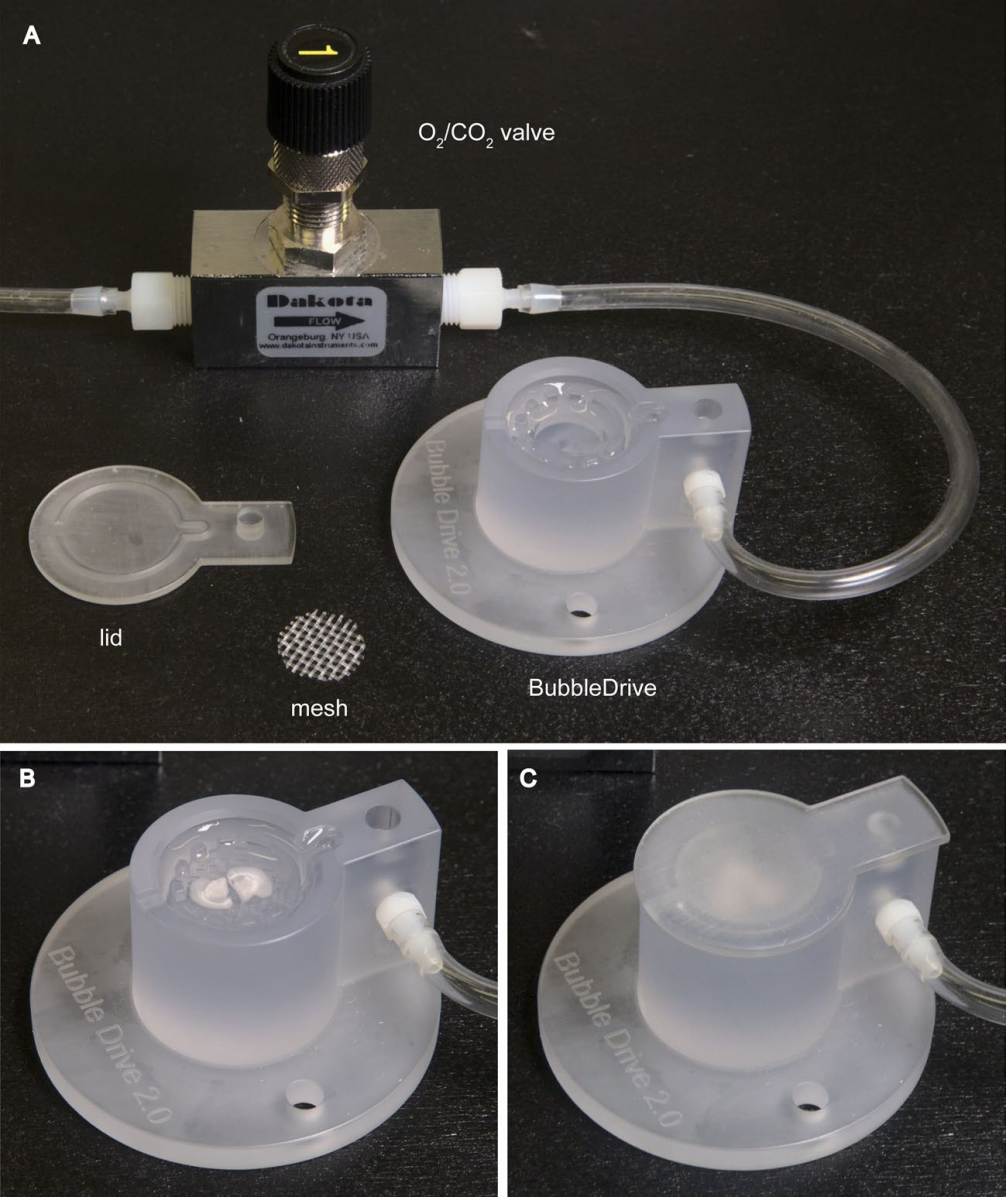
BubbleDrive in use. (**A**) A photograph of BubbleDrive with all its accessories: the gas valve and tubing, the mesh, and the (inverted) lid. The chamber with two (**B**) and three (**C**) coronal slices, shown without and with the lid in place, respectively.

**Figure 4:**
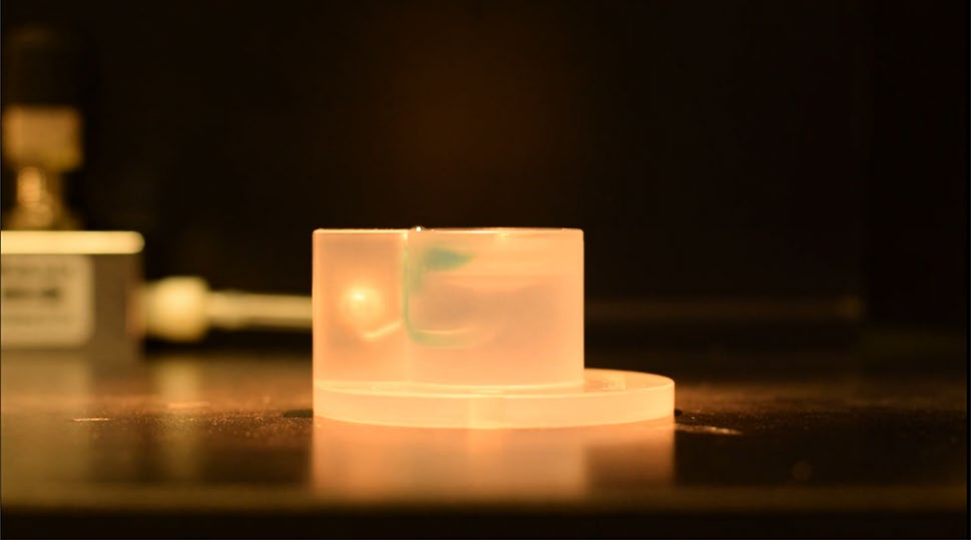
Liquid circulation in the BubbleDrive. The figure represents one frame of a Supplemental Video 1 which visualizes the movement of solution in the BubbleDrive using the Fast Green Dye. The dye was added at the center of the slice incubation area before turning on the gas flow. Notice the efficient mixing of the dye with the solution and the flow established immediately after turning on the gas flow.

The design principle of BubbleDrive uses the bubbles of gas rising vertically in a cylindrical conduit (similar to a chimney) to drive the ACSF circulation. The name given to this type of chamber, BubbleDrive, thus captures its operational principle. Figures 1, 2 and 3 show that except for the chimney section, the flows of the gas and the solution are conveniently separated. The gas is slowly injected at the bottom of the chimney, rises up in bubbles interrupted by short columns of the solution, and is subsequently released into the atmosphere under the chamber lid where it stays in contact with the solution above the slices until leaving via a small opening on the opposite side. The ACSF solution is brought from the basin under the slices and driven upwards along the chimney by the gas bubbles. Because there is a narrow vertical slot cut into the side of the chimney, the oxygenated liquid gradually escapes the upward pressure and flows sideways and downwards from the chimney into a circular channel shaped like a moat around the battlements surrounding the central slice chamber. This arrangement ensures that the solution enters the central slice chamber nearly uniformly and very gently from all sides. The circulation is then completed around the slices downward via a gradually narrowing duct to the lower part, where it is driven once more up into the chimney (see Figs. 2B and 4, which is available as a Supplemental Video 1).

One problem we encountered in the early prototype of the BubbleDrive was the surface tension occasionally holding the bubbles down in the chimney and restricting the flow. The final design therefore incorporates two low profile triangular ridges running up along the chimney wall, which slightly offset the bubbles from the surface of this wall and allow them to forge upwards without significant impediment. Another problem was with condensation on the chamber lid seeping outside. This was remedied by forming a triangular lip on the bottom of the lid which seals it and directs any condensate safely back into the circular moat.

The design was parametrically modeled in the Fusion 360 CAD system (Autodesk, San Rafael, CA). Its two parts, the chamber and the lid, were then exported into separate STEP files (Standard for the Exchange of Product Data, ISO 10303) and professionally manufactured (Proto Labs, Maple Plain, MN) by stereolithographic 3D printing which made very thin layering (50 $\mu$m) possible.

The chamber was manufactured from the Somos WaterShed XC 11122 material (DSM Functional Materials, Somos Material Group, Elgin, IL). According to the manufacturer’s data sheet, this material exhibits low moisture absorption (0.35%) important for this application, and relatively high tensile strength. Compared to typical injection-molded ABS plastic polymer values, Watershed has a slightly higher tensile strength (50.4 MPa) but suffers from a fairly low heat distortion temperature (50^∘^C). Some precautions are therefore necessary when washing and storing the BubbleDrive.

The mesh providing level support for the brain slices is resting on a circular ledge inside the chamber (Fig. 3). It is made from a sheet of stiff nylon mesh composed of threads about 0.5 mm thick (Small Parts, Inc., Miami Lakes, FL; catalog #CMN-1000-A). It is cut very slightly larger than the chamber’s inside diameter in order to introduce a small amount of friction holding it securely in place.

When using the BubbleDrive, it is important to be able to precisely regulate the incoming gas flow. We employed a metering needle valve designed for gasses and liquids with very low flow rates (Dakota Instruments, Orangeburg, NY; catalog #6AMV1101). This valve requires 16 turns to fully open and has negligible hysteresis, which makes the adjustments much easier than using the inconsistent tubing clamps typically found on the gas lines supplying traditional slice chambers.

### Validation in mouse brain slices

#### Structure of ECS and extracellular diffusion

**Figure 5:**
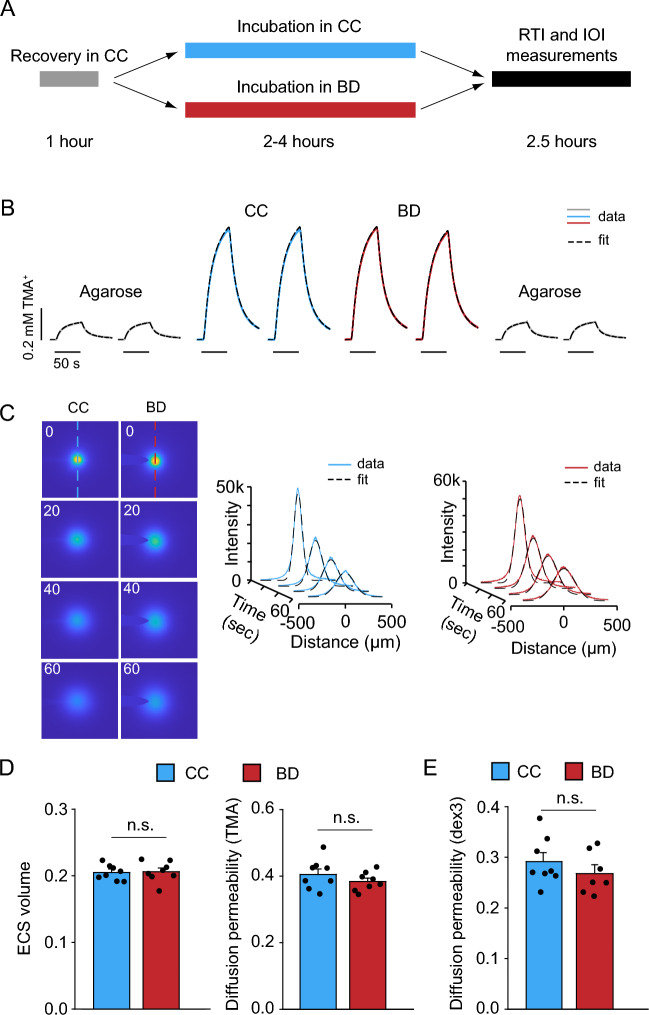
Structure of ECS and extracellular diffusion. (**A**) Timeline showing the incubation of slices in the BubbleDrive (BD) or conventional chamber (CC), and the ECS diffusion experiments. (**B**) Representative RTI experiment showing TMA^+^ diffusion records in the somatosensory cortex of slices incubated in BD and CC, and in dilute agarose gel before and after brain measurements. The horizontal bar on the x-axis shows the duration of TMA^+^ release for each record. For this experiment, the distance between the source and the detector microelectrodes was 130 $\mu$m and the average transport number was 0.318. For the slice from CC (an average from 2 records): $\alpha = 0.215$, $\theta = 0.431$, $\kappa = 0.009$ s^-1^; for the slice from BD (an average of 2 records): $\alpha = 0.214$, $\theta = 0.479$, $\kappa = 0.007$ s^-1^. (**C**, left) Representative dex3 diffusion records in the somatosensory cortex of slices incubated in each chamber. (**C**, right) Change with time in the intensity profiles along the highlighted axis. $\theta = 0.312$ for BD and 0.334 for CC. (**D**) Summary of the ECS volume fraction and diffusion permeability data for TMA^+^. Data from each slice (circles), mean values (bars) and SEM (error bars) are shown. (**E**) Summary of dex3 diffusion permeability data. Data from each slice (circles), mean values (bars) and SEM (error bars) are shown.

We began by quantifying the values of ECS volume fraction and diffusion permeability in two groups of mouse brain slices (see the Methods section for definitions of these parameters). One group was incubated for 3–4 hours in the BubbleDrive and the other group was incubated in a submersion conventional chamber (CC). To this end, we employed the Real-Time Iontophoretic (RTI) method with a small extracellular probe tetramethylammonium (TMA^+^, 74 MW) to quantify the volume fraction $\alpha$ and the diffusion permeability $\theta$. The timeline of the RTI experiment is shown in Fig. 5A. Figure 5B shows representative diffusion curves (i.e. the concentration of TMA^+^ at the detector as a function of time) along with fitted theoretical curves. The fitting procedure yielded the ECS volume fraction $\alpha$, diffusion permeability $\theta$, and non-specific clearance $\kappa$. Figure 5D shows the summary of all results. There were no significant differences between the chambers in either parameter. ECS volume fraction was $0.205 \pm 0.004$ (mean ± SEM; $n = 8$) in the slices incubated in CC and $0.206 \pm 0.005$ ($n = 8$) in the slices incubated in BubbleDrive (t-test, $p = 0.848$). Diffusion permeability was $0.405 \pm 0.016$ in the slices incubated in CC and $0.384 \pm 0.010$ in the slices incubated in BubbleDrive (t-test, $p = 0.265$). Non-specific clearance was $0.012 \pm 0.001$ s^-1^ in the slices incubated in CC and $0.009 \pm 0.001$ s^-1^ in the slices incubated in BubbleDrive (t-test, $p = 0.129$).

We also evaluated extracellular diffusion of a fluorescently labeled dextran macromolecule (dex3, MW 3000) using Integrative Optical Imaging (IOI) technique. The timeline of the IOI experiment is shown in Fig. 5A. Representative diffusion data sets for both groups are presented in Fig. 5C. The diffusion permeability $\theta$ for dex3 was compared between the slices incubated in CC vs. BubbleDrive. These were found to be similar to each other: $0.291 \pm 0.017$ in CC ($n = 8$) and $0.268 \pm 0.016$ in BubbleDrive (Fig. 5E; $n = 7$, t-test, $p = 0.327$).

Taken together, these data show that the ECS structural parameters and extracellular diffusion are unaltered by incubation in the BubbleDrive.

#### Excitatory synaptic transmission

**Figure 6:**
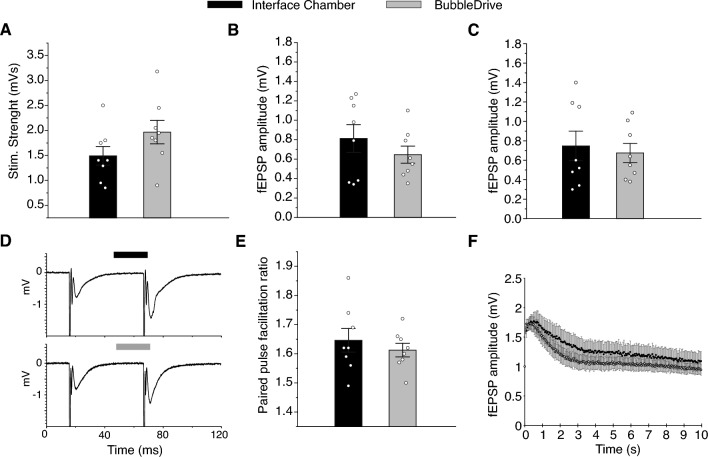
Synaptic transmission, excitability, paired-pulse facilitation (PPF) and 20 Hz stimulation in stratum radiatum. (**A**) Stimulation strengths necessary to elicit a pre-volley of 1 mV in amplitude. (**B**) Field EPSP amplitude (fEPSP amplitude) as a function of the same pre-volley amplitude. (**C**) The fEPSP amplitude necessary to elicit a just detectable population spike on the fEPSP. (**D**) Example fEPSPs from slices incubated in the two incubation chambers. A mean of six consecutive synaptic responses which elicited synaptic stimulation. (**E**) Paired pulse facilitation ratio at an interstimulus interval of 50 ms. (**F**) Dynamic fEPSP amplitude as a result of 20 Hz stimulation for 10 s. Data are shown as mean ± SEM.

To reveal potential differences in excitatory synaptic transmission and cell excitability as a result of incubation conditions, we recorded synaptic activation in the apical dendritic layer (stratum radiatum) in the CA1 region of hippocampal slices from mice. The stimulation strength necessary to elicit a pre-volley (compound action potential of the axons that are activated with a stimulating electrode placed approximately 200 $\mu$m away from the recording electrode) of 1.0 mV in amplitude was similar in both chambers (Fig. 6A; $1.5 \pm 0.2$ mVs, $n = 8$ slices compared to $2.0 \pm 0.3$ mVs, $n = 8$ slices, incubated in an interface chamber vs. the BubbleDrive, respectively; t-test, $p = 0.14$). We also found the field excitatory postsynaptic potential (fEPSP) as a function of the same pre-volley amplitude to be similar in the two groups (Fig. 6B; $0.81 \pm 0.15$ mV, $n = 8$ slices; $0.65 \pm 0.09$ mV, $n = 8$ slices, incubated in an interface chamber vs. the BubbleDrive, respectively; t-test, $p = 0.34$), suggesting an unchanged excitatory synaptic transmission. Furthermore, postsynaptic excitability measured as fEPSPs necessary for generating a population spike was not significantly different between the two groups (Fig. 6C; $0.75 \pm 0.16$ mV, $n = 8$ slices; $0.68 \pm 0.11$ mV, $n = 8$ slices, incubated in the interface vs. the BubbleDrive chambers, respectively; t-test, $p = 0.70$).

To further characterize excitatory synaptic transmission in the hippocampal CA1 region, we measured paired-pulse facilitation (PPF), which is a short-lasting form of synaptic plasticity primarily attributed to changes in presynaptic Ca^2+^ homeostasis. PPF was again similar in the two groups (Fig. 6D and E; $1.65 \pm 0.04$, $n = 8$ slices; $1.61 \pm 0.03$, $n = 8$ slices, incubated in the interface chamber vs. the BubbleDrive, respectively; t-test, $p = 0.49$).

Finally, we tested the ability of slices to respond to a prolonged period of synaptic stimulation (20 Hz for 10 s) as a measure of glutamate release/reuptake and presynaptic vesicle dynamics. No clear differences in the dynamic synaptic response during the 20 Hz stimulation were evident between the two incubation conditions (Fig. 6F).

#### Astrocytic Ca^2+^ signal responses to synaptic stimulation

**Figure 7:**
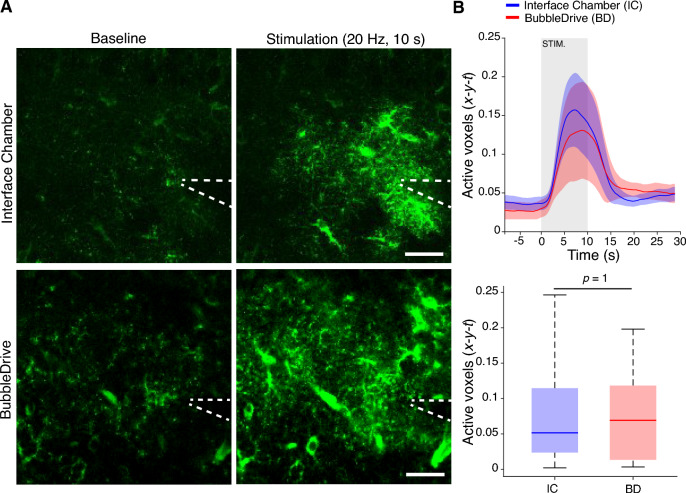
Astrocytic Ca^2+^ signaling responses to electrophysiological stimulation in hippocampal brain slices. (**A**) Representative mean projection of the baseline period and the stimulation period. Stippled line denotes placement of stimulation electrode. Scale bar 20 $\mu$m. (**B**, top) Mean active x-y-t voxels (detected with the ROA method) with astrocytic Ca^2+^ signals over time from slices incubated in an interface chamber and in the BubbleDrive. (**B**, bottom) Mean percentage of active x-y-t voxels (detected with the ROA method) in the stimulation period. Statistical analysis using Wilcoxon Rank Sum test.

Astrocytes are known to be vulnerable to tissue preparation and sensitive to slice preparation and incubation. For instance, there are rapid ultrastructural changes, expression of markers of reactive gliosis, and mitochondria and receptor expressions, all found after slice preparation and incubation in a submersion-type chamber^2^. For these reasons we typically use an interface chamber for storing slices before experiments involving astrocyte studies where normal ultrastructure and glycogen granules reappear within a typical resting period before data recording commences^3^. In the following we compare incubation in an interface-type resting chamber with the BubbleDrive, followed in both cases by two-photon imaging in a submersion-type chamber. We prepared acute hippocampal slices expressing the genetically encoded Ca^2+^ sensor GCaMP6f under the GFAP promoter and recorded responses in the stratum radiatum of the CA1 region to a 10 s, 20 Hz electrophysiological stimulation of the Schaffer collaterals. As reported previously^4,5^, this stimulation protocol elicits robust astrocytic Ca^2+^ increases. Time-series data were analyzed with an ultrasensitive signal detection algorithm termed the ROA algorithm^6,7^, where signal is detected in a pixel-by-pixel fashion based on the noise statistics of the pixel over time. There was no difference in Ca^2+^ response (ROA density = fraction of the field of view active) between the two groups (Fig. 7; $n = 12$ slices for CC and $n = 7$ slices for BubbleDrive; $p = 1$ when comparing average activation in the stimulation period using Wilcoxon rank-sum test), demonstrating preserved astrocyte responses in the BubbleDrive.

### Application: incubation of brain slices with hyaluronidase

**Figure 8:**
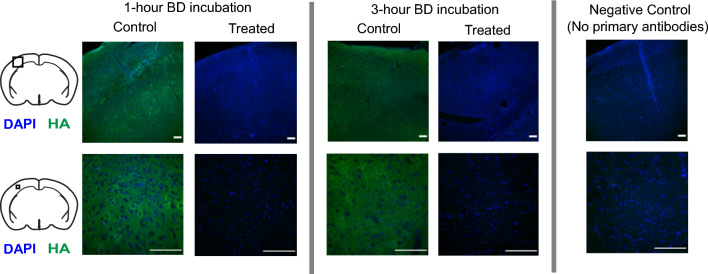
Incubation of brain slices with hyaluronidase. Representative confocal images of immunohistochemistry in somatosensory cortex showing HA in green and cell nuclei in blue (DAPI staining) after 1 hour and 3 hours of incubation with hyaluronidase in BubbleDrive. Loss of HA intensity (green) due to enzymatic cleavage is apparent at both incubation times when compared to control slices. Cleavage of HA at the center of the slice even with 1 hour incubation in BubbleDrive shows a time-efficient penetration of the enzyme into the tissue. Scale bar 100 $\mu$m.

BubbleDrive was designed in order to incubate brain slices with the least amount of incubation solution. We employed the enzyme hyaluronidase that cleaves HA in the extracellular space and verified effectiveness of this treatment by immunohistochemistry. Hyaluronidase was added to ACSF at a concentration of $85 \pm 5$ U/mL. Different incubation times were tested in order to see how quickly the enzyme penetrated into the tissue and showed its effect. We know from previous publications that 2-hour incubation with the bath concentration of 70 U/mL was sufficient to remove most of HA in brain slices^8^. Representative confocal images of immunohistochemistry for the shortest and the longest incubation times are shown in Fig. 8. In comparison to control sections (n = 3), after 1 hour hyaluronidase incubation (n = 3), the intensity of HA in the slices decreased profoundly, showing that most of the HA had already been cleaved by the end of 1 hour. The intensity remained low for slices incubated for longer time (1.5 and 3 hours; n = 4), as expected. We conclude that incubation of brain slices with hyaluronidase in BubbleDrive is effective in cleaving HA. BubbleDrive can thus be used for a variety of applications that involve incubation with expensive chemicals.

## Discussion

In this paper, we have introduced BubbleDrive, a new low-volume 3D-printed incubation chamber. We thoroughly validated its ability to maintain health of the incubated slices in a series of experiments. The BubbleDrive is able to maintain ECS characteristics as well as neuronal and astrocytic functions. We also employed it in an experiment where the slices required incubation with an expensive enzyme for more than 1 hour. Using the BubbleDrive incubation chamber drastically reduced the cost of this experiment.

Lack of commercially available low-volume incubation chambers that we needed for our project spurred on the development of BubbleDrive. Our search for low-volume incubation chambers revealed that Scientific Systems Design Inc (Milton, Ontario, Canada) produces two types of low-volume Brain Slice Keepers (https://scisys.info/brain-slice-keepers). Brain Slice Keeper 2 uses a minimum of 5 mL of incubation media while Brain Slice Keeper 6 uses 4 mL of incubation media in each of its six wells. Both Keeper models accommodate 1–2 brain slices. Similarly to BubbleDrive, circulation of the incubation media is driven by a stream of gas bubbles. However, the Keeper’s design forces the entire flow volume upwards to the surface while BubbleDrive’s design distributes the delivered liquid along the length of a slot in a vertical chimney, then around the chamber perimeter and through the notched openings, before finally flowing over the slices. A small volume of saturated gas above the liquid surface reduces evaporation which could otherwise result in altered concentrations over time. Overall, BubbleDrive is more efficient than any of the Brain Slice Keepers, requiring a smaller volume of the incubation media (1.5–2 mL vs. 4–5 mL) while accommodating more slices (3 vs. 1–2). Finally, the BubbleDrive design is freely available to any researcher upon request, facilitating 3D printing or possible further design modifications, providing such modifications are also made freely available to the research community.

Literature search also yielded two incubation chambers designed in-house. The first is a simple lollipop-shaped aerator designed by Meitzen lab^1^. The aerator served as a source of oxygen while the nylon mesh stretched across its circular part provided mechanical support for the slice. The aerator could thus be introduced inside the well of a standard multi-well plate, making each well into a low volume incubation chamber. Each well, filled with 2 mL of incubation media, could accommodate 1–2 brain slices. However, the cited article states that one change of the solution in the well during the incubation was needed to improve the health of the slices. This effectively increases the total volume of solution required for the incubation. In addition, gas bubbles were delivered into incubation solution in close proximity to the mesh supporting the brain slices. This arrangement could lead to mechanical disturbance of the slices, possibly necessitating lower gas flow and slower liquid circulation. The gas bubbles could also become trapped by the mesh underneath the slice, which would compromise the slice health. BubbleDrive is more efficient than the aerator because it requires a smaller total ACSF volume (1.5–2 mL vs. 4 mL) while accommodating more slices (3 vs. 1–2). One advantage of the aerator over the BubbleDrive is its low cost.

Another low-volume incubation chamber we encountered used similar strategy^9^. It featured a plate with 6 wells and a cell strainer basket with 100 $\mu$m pore size inside each well. The solution around and below the well was bubbled with carbogen using a 22-gauge needle placed between the wall of the well and the basket. The base of the basket held the slices and also allowed for mixing of the solution above and below the basket while preventing mechanical damage to the slices. This arrangement allowed for incubation of slices in parallel because of multiple wells. However, each well used a minimum of 3 ml of incubation solution, which is approximately twice the amount of solution needed for BubbleDrive. Two advantages of this chamber over BubbleDrive are its low cost and the possibility of parallel incubation of slices with different media.

Other low-volume incubation chambers that we have found in our search employed larger volumes of the flowing incubation solutions. Thus, although the incubation chamber itself may have a small volume, the amount of needed incubation solution was still high and therefore requiring high amount of expensive enzymes^10^.

It is worth noting that in the experiments with astrocytic networks, BubbleDrive was successfully compared to the interface chamber usually employed in such studies because they tend to preserve the astrocytes better than conventional submersion chambers. Even though BubbleDrive is a submersion chamber, its flow is better defined and more consistent than in the conventional chamber, which may reduce stagnant layers at the slice surface. Its carbogen delivery is also less limited by the necessity to reduce the bath churning. It is thus likely that BubbleDrive could be used in other experiments involving astrocytes, with the advantage of eliminating the drawbacks of the interface chambers, especially the undesirable pronounced vertical gradient of the slice environment.

As is, BubbleDrive can accommodate up to three mouse coronal slices cut from one hemisphere. Fewer slices would be accommodated if different sectioning planes were used, e.g. sagittal or horizontal, or if slices were prepared from larger brains, e.g. from a rat brain. In such cases, we sometimes use multiple BubbleDrives in parallel. Alternatively, BubbleDrive could be scaled up but that would make the resulting chamber less universally useful.

While BubbleDrive was intended strictly for slice incubation, the encouraging results above suggest that its use may possibly be extended to a recording chamber. Additional openings in the lid could accommodate microelectrodes in electrophysiological experiments, or an objective of a vertical epifluorescent microscope. A set of lids for various such purposes could easily be made. However, we have not yet tested any such usage.

In conclusion, we report the design, validation and application of a low-volume BubbleDrive incubation chamber. Although we used mouse brain slices, the chamber can be employed to maintain brain slices obtained from other species. It would be relatively easy to scale up the geometry if a larger piece of tissue were to be incubated, e.g. an isolated turtle cerebellum, but any modification would require validation because the solution flow pattern or the oxygenation could inadvertently be altered. Another simple and useful modification may consist in addition of a heat exchanger channel circulating warm or cold water, should the experiment necessitate incubation at temperatures higher or lower than the room temperature. Alternatively, the device can be partially submerged in a bath.

## Methods

### Animals

All methods involving animals were approved by the local Institutional Animal Care and Use Committee at the SUNY Downstate Health Sciences University or by the Norwegian Food Safety Authority (project number 27102). All experiments were performed in accordance with the guidelines and regulations of these entities. The authors also complied with the ARRIVE guidelines. A total of 13 C57BL/6 mice (males and females 2–4.5 months old) were used at SUNY Downstate, and 11 mice of the same strain at University of Oslo. All animals were group-housed, maintained on a 12-hour light-dark cycle and fed ad libitum prior to brain slice preparation.

### Slice preparation

At SUNY Downstate Health Sciences University, brain slices were prepared from mice anesthetized with urethane (U2500, Sigma, Burlington, MA) solution made in 150 mM NaCl (single dose of 1.8–1.9 g/kg body weight of animal, i.p.). The animal was then decapitated and the brain was extracted and submerged in artificial cerebrospinal fluid (ACSF) containing (in mM) 124 NaCl, 5 KCl, 26 NaHCO_3_, 1.25 NaH_2_PO_4_, 10 D-Glucose, 1.3 MgCl_2_, and 1.5 CaCl_2_ pre-cooled to 4^∘^C. Vibrating microtome (Leica VT1000S; Leica Microsystems GmbH, Wetzlar, Germany) was used to make 400 $\mu$m thick coronal slices of one brain hemisphere. ACSF was continuously bubbled with 95% O_2_ and 5% CO_2_ mixture. Slices were transferred to a CC and let to recover for 1 hour at room temperature. RTI experiments also required the addition of 0.5 mM of TMA-Cl to ACSF.

At University of Oslo, adult mice were given an overdose of Isoflurane (Baxter, Deerfield, IL). Their brains were gently removed from the skull and placed in a beaker filled with artificial cerebrospinal fluid (ACSF at 20^∘^C, bubbled with 95% O_2_ and 5% CO_2_ gas mixture) and containing (in mM): 124 NaCl, 2 KCl, 1.25 KH_2_PO_4_, 2 MgSO_4_, 2 CaCl_2_, 26 NaHCO_3_ and 12 glucose. After carefully removing the brainstem, a small brain tissue block consisting of both cortex and hippocampus (dorsal/middle) was fixed to a cutting stage with a tiny drop of cyanoacrylate. Transverse slices (400 $\mu$m) were then cut with a vibroslicer at room temperature (Leica S1200, sapphire blade from Ted Pella, Inc., Redding, CA) and kept in the cutting chamber until all the slices were cut (up to 20 min). Slices were then transferred to either an interface chamber exposed to humidified gas at 30^∘^C or the BubbleDrive at 28.5^∘^C. To maintain these temperatures, both chambers were partially submerged in a water bath set at 32^∘^C.

### Real-time iontophoresis

We used the Real-Time Iontophoretic (RTI) method^11,12^ with TMA^+^ as an extracellular molecular probe to extract two structural parameters of ECS: the ECS volume fraction, $\alpha = V_{\textrm{ESC}} /V_{\textrm{tissue}}$, and the diffusion permeability, $\theta = \frac{D^\star }{D_{\textrm{free}}}$, where $D^\star$ is the effective diffusion coefficient of TMA^+^ in brain tissue and $D_{\textrm{free}}$ is the free diffusion coefficient of the same substance^13^. In RTI experiments, TMA^+^ was iontophoretically released from a glass microelectrode and detected by an ion-selective microelectrode (ISM) positioned 100–130 $\mu$m away. All RTI measurements were performed in the somatosensory neocortex at the depth of 200 $\mu$m below the surface of 400 $\mu$m thick slices.

TMA^+^ signal was recorded using the Venda program written in-house in MATLAB (MathWorks, Natick, MA). Acquired records were fitted with an appropriate solution of the diffusion equation^11,12^ using the program Valter written also in-house in MATLAB. Diffusion records from the dilute agarose gel (0.3% w/v in 150 mM NaCl, 0.5 mM TMA-Cl; NuSieve GTG Agarose, cat #50081; Lonza, Rockland, ME) provided the transport number $n_t$ of the iontophoretic microelectrode and the free diffusion coefficient $D_{\textrm{free}}$ of TMA^+^, which were then used to determine $\alpha$ and $\theta$ from brain records.

### Integrative optical imaging (IOI)

IOI was used to quantify the extracellular diffusion of a fluorescently labeled dextran macromolecule in the brain extracellular space. The technique was explained in detail previously^14,15^. Briefly, fluorescent macromolecule was pressure-injected from a single barrel glass micropipette into the brain tissue or dilute agarose gel and a sequence of images was taken using a charged-coupled device (CCD) camera attached to an epifluorescence microscope. The distribution of the fluorescence signal was fitted to a diffusion equation to obtain an estimate of the effective diffusion coefficient $D^*$ and the free diffusion coefficient $D_{\textrm{free}}$ of that molecule in the brain tissue and dilute agarose gel preparation, respectively. The diffusion permeability $\theta$ for the dextran macromolecule was then calculated.

### Electrophysiology

After 2–3 hours of incubation in the two different chambers, the BubbleDrive and an interface chamber, the slices were then transferred to a recording chamber (Slice mini chamber I, Luigs &Neumann, Ratingen, Germany) submerged at 30^∘^C. Extracellular synaptic responses were recorded by one glass electrode (approx. 2 M$\Omega$) filled with ACSF, placed in stratum radiatum. Orthodromic synaptic stimuli were given with a similar glass electrode separated by 200 $\mu$m. Both electrodes were placed approximately 50 $\mu$m deep into the slice. Signals were captured, recorded and analyzed using the Multiclamp 700B, Digidata 1440A and P-Clamp 10 (Molecular Devices, San Jose, CA). Four types of experiments were performed: *1. Synaptic transmission.* The stimulus strength was set to elicit a 1 mV presynaptic fiber volley and the field excitatory postsynaptic potential (fEPSP) amplitude was measured. *2. Paired-pulse facilitation.* A similar approach was used to elicit paired-pulse responses (50 ms interstimulus interval, the two stimuli being equal in strength). The stimulus strength was set just below eliciting a population spike on the second pulse. *3. Synaptic excitability.* The excitability was measured as the fEPSP amplitude needed to give a visible positive deflection on the fEPSP, indicating a population spike. *4. Glutamate dynamics.* The release and recycling of glutamate vesicles was indirectly studied by prolonged synaptic stimulation (20 Hz for 10 seconds) and recording the corresponding fEPSP as a proxy for glutamate release.

### Calcium imaging in astrocytes

In this set of experiments 70 nL of virus (GFAP-GCaMP6f) was injected bilaterally into the hippocampus (coordinates relative to the Bregma were: anterioposterior -2 mm, mediolateral 1.5 mm and dorsoventral -1.6 mm). After 4 weeks, hippocampal slices were prepared as described above and the astrocytes ability to respond with Ca^2+^ transients to synaptic stimulation was studied. Stimulation strength was adjusted to give a detectable population spike on the fEPSP. After 2 minutes of image recording the axons were stimulated at 20 Hz for 10 seconds, and the astrocytic Ca^2+^ response was recorded by two-photon microscopy (model Ultima; Prairie Technologies, Middleton, WI) with a 25X water-immersion objective with a numerical aperture of 1.05 (XLPN 25WMP, Olympus America, Melville, NY) using a Chameleon Vision II laser (Coherent, Santa Clara, CA) with 920 nm laser pulses for excitation.

To analyze the image time series, fluorescence images were imported into MATLAB (MathWorks). Astrocytic Ca^2+^ activity was detected with the so-called “region-of-activity” method^6^. In short, Ca^2+^ signals were detected on a pixel-by-pixel basis using noise-based thresholding. Plots and statistics were obtained in MATLAB.

### Enzymatic removal of hyaluronan (HA) and immunohistochemistry

To remove HA from ECS, slices in BubbleDrive were incubated with ACSF containing hyaluronidase from *Streptomyces hyalurolyticus* (H1136, Sigma) at a concentration of $85 \pm 5$ U/mL. After 1 hour, 1.5 hours and 3 hours of incubation, hyaluronidase-treated slices from the BubbleDrive and matching control slices from the CC were collected for immunohistochemistry experiment.

Collected brain slices (control and hyaluronidase-treated) were fixed overnight using Formalin solution (S25689, Thermo Fisher Scientific, Waltham, MA) at 4^∘^C. Next day, the slices were washed 6 times with 1X PBS and cut into 40 $\mu$m thick sections with vibratome (Leica VT1200). One section from the center of each 400 $\mu$m slice was used for comparison between the control and hyaluronidase-treated groups.

All sections were first washed (0.3% Triton X-100 in 1X PBS) and then incubated with blocking solution (0.1% Triton X-100 + 1% BSA in 1X PBS) for 1 hour at room temperature. Next, sections were incubated with Biotinylated Hyaluronan Binding Protein (Biotin-HABP; 385911, Millipore Sigma, Burlington, MA; diluted 1:100 with blocking solution) overnight at 4^∘^C. The sections were then washed with washing buffer 6 times at room temperature. Two additional sections taken from untreated tissue, which acted as a control for non-specific binding, were incubated with the blocking solution only (without Biotin-HABP) in this step. All sections were incubated with Streptavidin-Alexa Fluor 488 (S11223, Thermo Fisher Scientific; diluted 1:500 with blocking solution) at room temperature for 4 hours, washed with a washing buffer and mounted onto Superfrost Plus microscope slides (22-037-246, Thermo Fisher Scientific) with ProLong Gold antifade mounting medium with 4’,6-diamidino-2-phenylindole (DAPI; P36931, Thermo Fisher Scientific) to stain for DNA in cell nuclei. This protocol was modified from a previously published work^16^. Fluoview FV1000 confocal scanning microscope (Olympus America) and Zeiss LSM800 Upright confocal laser scanning microscope (Zeiss, Dublin, CA) were used to visualize the sections at 10X and 40X magnifications.

### Statistics

Except for the calcium imaging results, all data are presented as mean $\pm$ SEM and *n* denotes the number of slices. Statistical analyses were performed using SigmaStat (Systat Software Inc., San Jose, CA). Normality and equal variances of data sets were tested first in order to determine whether a parametric or non-parametric version (Wilcoxon Rank Sum test) of a t-test was needed. Significance level was set at $p < 0.05$.

For RTI and IOI experiments, one data point represents the average of several records from one slice. For electrophysiology experiments, one data point represents a maximum amplitude measurement done on a mean sweep from six consecutive EPSP sweeps from one slice at a given stimulation strength. Stimulation strength was calculated as voltage $\times$ duration (mV $\times$ ms).

For the calcium imaging experiments, one data point represents the mean number of active voxels for the first 80 s from the start of the stimulation. Data are presented as median and a corresponding 25th to 75th percentile range.

For immunohistochemistry experiments, one 40 $\mu$m section was selected from each 400 $\mu$m slice and several images were taken for each section. The image intensity was used to qualitatively draw conclusions about enzymatic cleavage of HA.

### Supplementary Information

Supplementary Legends.

Supplementary Video 1.

## Data Availability

Data supporting our results and the STEP files containing the BubbleDrive design are available from the authors upon reasonable request for non-commercial use, providing that any design modifications are also made available to the research community. Contact S.H. (sabina.hrabetova@downstate.edu).

## References

[1] Dorris DM, Hauser CA, Minnehan CE, Meitzen J (2014). An aerator for brain slice experiments in individual cell culture plate wells. J. Neurosci. Methods.

[2] Takano T (2014). Rapid manifestation of reactive astrogliosis in acute hippocampal brain slices. Glia.

[3] Fiala JC (2003). Timing of neuronal and glial ultrastructure disruption during brain slice preparation and recovery in vitro. J. Comp. Neurol..

[4] Tang W (2015). Stimulation-evoked Ca^2+^ signals in astrocytic processes at hippocampal CA3-CA1 synapses of adult mice are modulated by glutamate and ATP. J. Neurosci..

[5] Szokol K (2015). Augmentation of Ca^2+^ signaling in astrocytic endfeet in the latent phase of temporal lobe epilepsy. Front. Cell. Neurosci..

[6] Bjørnstad DM (2021). Begonia-a two-photon imaging analysis pipeline for astrocytic Ca^2+^ signals. Front. Cell. Neurosci..

[7] Bojarskaite L (2020). Astrocytic Ca^2+^ signaling is reduced during sleep and is involved in the regulation of slow wave sleep. Nat. Commun..

[8] Kochlamazashvili G (2010). The extracellular matrix molecule hyaluronic acid regulates hippocampal synaptic plasticity by modulating postsynaptic L-type Ca^2+^ channels. Neuron.

[9] Hupp S, Tomov NS, Bischoff C, Baronti D, Iliev AI (2022). Easy to build cost-effective acute brain slice incubation system for parallel analysis of multiple treatment conditions. J. Neurosci. Methods.

[10] Dondzillo A (2015). A recording chamber for small volume slice electrophysiology. J. Neurophysiol..

[11] Nicholson C, Phillips JM (1981). Ion diffusion modified by tortuosity and volume fraction in the extracellular microenvironment of the rat cerebellum. J. Physiol..

[12] Odackal J (2017). Real-time iontophoresis with tetramethylammonium to quantify volume fraction and tortuosity of brain extracellular space. J. Vis. Exp..

[13] Hrabe J, Hrabetova S, Segeth K (2004). A model of effective diffusion and tortuosity in the extracellular space of the brain. Biophys. J..

[14] Nicholson C, Tao L (1993). Hindered diffusion of high molecular weight compounds in brain extracellular microenvironment measured with integrative optical imaging. Biophys. J..

[15] Hrabe J, Hrabetova S (2019). Time-resolved integrative optical imaging of diffusion during spreading depression. Biophys. J..

[16] Arranz AM (2014). Hyaluronan deficiency due to Has3 knock-out causes altered neuronal activity and seizures via reduction in brain extracellular space. J. Neurosci..

