# Author Correction: BubbleDrive, a low-volume incubation chamber for acute brain slices

**DOI:** 10.1038/s41598-024-52441-5

**Published:** 2024-01-23

**Authors:** Aditi Naik, Vidar Jensen, Cecilie Bugge Bakketun, Rune Enger, Sabina Hrabetova, Jan Hrabe

**Affiliations:** 1 Department of Cell Biology, State University of New York Downstate Health Sciences University, Brooklyn, NY USA; 2 Neural and Behavioral Science Graduate Program, State University of New York Downstate Health Sciences University, Brooklyn, NY USA; 3 Letten Centre, Division of Anatomy, Department of Molecular Medicine, Institute of Basic Medical Sciences, University of Oslo, Oslo, Norway; 4 Department of Neurology, Oslo University Hospital, Oslo, Norway; 5 The Robert F. Furchgott Center for Neural and Behavioral Science, State University of New York Downstate Health Sciences University, Brooklyn, NY USA; 6 Translational Neuroscience Laboratories, Center for Biomedical Imaging and Neuromodulation, Nathan S. Kline Institute for Psychiatric Research, Orangeburg, NY USA

Correction to: *Scientific Reports* 10.1038/s41598-023-45949-9, published online 16 November 2023

The original version of this Article erroneously named the distributor of the Brain Slice Keeper system as the manufacturer. This has now been corrected and in the Discussion section,

“Our search for low-volume incubation chambers revealed that AutoMate Scientific (Berkeley, CA, USA) produces two types of low-volume Brain Slice Keepers (https://www.autom8.com/product-category/brain-slice-tissue-chambers/brain-slice-incubators).”

now reads:

“Our search for low-volume incubation chambers revealed that Scientific Systems Design Inc (Milton, Ontario, Canada) produces two types of low-volume Brain Slice Keepers (https://scisys.info/brain-slice-keepers).”

