# Supplementary material for: BubbleDrive, a low-volume incubation chamber for acute brain slices
Source: Sci Rep. 2023 Nov 16;13:20005. doi: 10.1038/s41598-023-45949-9 (PMC10654715; doi:10.1038/s41598-023-45949-9)
Supplement: Supplementary file 1 — Supplementary Legends. [file 41598_2023_45949_MOESM1_ESM.pdf]

BubbleDrive, a low-volume incubation chamber for acute brain slices

Aditi Naik[1,2,+], Vidar Jensen[3,+], Cecilie Bugge Bakketun[3,4], Rune Enger[3,*], Sabina Hrabetova[1,5,*], and Jan Hrabe[6,1,*]

[1]Department of Cell Biology, State University of New York Downstate Health Sciences University, Brooklyn, NY, USA
[2]Neural and Behavioral Science Graduate Program, State University of New York Downstate Health Sciences University, Brooklyn, NY, USA
[3]Letten Centre, Division of Anatomy, Department of Molecular Medicine, Institute of Basic Medical Sciences, University of Oslo, Oslo, Norway
[4]Department of Neurology, Oslo University Hospital, Oslo, Norway
[5]The Robert F. Furchgott Center for Neural and Behavioral Science, State University of New York Downstate Health Sciences University, Brooklyn, NY, USA
[6]Translational Neuroscience Laboratories, Center for Biomedical Imaging and Neuromodulation, Nathan S. Kline Institute for Psychiatric Research, Orangeburg, NY, USA
[*]Corresponding autors, rune.enger@medisin.uio.no, sabina.hrabetova@downstate.edu, jan.hrabe@nki.rfmh.org
[+]These authors contributed equally to this work.

Supplemental Material

Supplementary video 1

Liquid circulation in the BubbleDrive. This video visualizes the movement of solution in the BubbleDrive using the Fast Green Dye. The dye was added at the center of the slice incubation area before turning on the gas flow. Notice the efficient mixing of the dye with the solution and the flow established immediately after turning on the gas flow.
